# Tanshinone IIA Stimulates Cystathionine γ-Lyase Expression and Protects Endothelial Cells from Oxidative Injury

**DOI:** 10.3390/antiox10071007

**Published:** 2021-06-23

**Authors:** Qiaojing Yan, Zhimin Mao, Jingru Hong, Kun Gao, Manabu Niimi, Takahiko Mitsui, Jian Yao

**Affiliations:** 1 Division of Molecular Signaling, Department of the Advanced Biomedical Research, Interdisciplinary Graduate School of Medicine, University of Yamanashi, Chuo 409-3898, Japan; g19dim03@yamanashi.ac.jp (Q.Y.); maoxyx@gmail.com (Z.M.); lulujane0130@gmail.com (J.H.); kungao@njucm.edu.cn (K.G.); 2 Division of Molecular Pathology, Department of the Advanced Biomedical Research, Interdisciplinary Graduate School of Medicine, University of Yamanashi, Chuo 409-3898, Japan; manabun@yamanashi.ac.jp; 3 Department of Urology, Interdisciplinary Graduate School of Medicine and Engineering, University of Yamanashi, Chuo 409-3898, Japan; tmitsui@yamanashi.ac.jp

**Keywords:** tanshinone IIA, cystathionine γ-lyase, oxidative cell injury, hydrogen sulfide, protein kinase A, endothelial cells

## Abstract

Tanshinone IIA (Tan IIA), an active ingredient of Danshen, is a well-used drug to treat cardiovascular diseases. Currently, the mechanisms involved remain poorly understood. Given that many actions of Tan IIA could be similarly achieved by hydrogen sulfide (H_2_S), we speculated that Tan IIA might work through the induction of endogenous H_2_S. This study was to test this hypothesis. Exposure to endothelial cells to Tan IIA elevated H_2_S-synthesizing enzyme cystathionine γ-Lyase (CSE), associated with an increased level of endogenous H_2_S and free thiol activity. Further analysis revealed that this effect of Tan IIA was mediated by an estrogen receptor (ER) and cAMP signaling pathway. It stimulated VASP and CREB phosphorylation. Inhibition of ER or PKA abolished the CSE-elevating effect, whereas activation of ER or PKA mimicked the effect of Tan IIA. In an oxidative endothelial cell injury model, Tan IIA potently attenuated oxidative stress and inhibited cell death. In support of a role of endogenous H_2_S, inhibition of CSE aggerated oxidative cell injury. On the contrary, supplement of H_2_S attenuated cell injury. Collectively, our study characterized endogenous H_2_S as a novel mediator underlying the pharmacological actions of Tan IIA. Given the multifaceted functions of H_2_S, the H_2_S-stimulating property of Tan IIA could be exploited for treating many diseases.

## 1. Introduction

Danshen is derived from the dried root of rhizome of Salviae miltiorrhizae Bge. In Chinese traditional medicine, Danshen activates blood circulation and reverses blood stasis. Tanshinone IIA (Tan IIA) is a crucial active ingredient of Danshen. It is a derivative of phenanthrenequinone with a broad range of pharmacological actions on vascular systems, including vasodilation, anticoagulation, antiinflammation, and antioxidation. It has been widely used to treat many cardiovascular diseases, including hypertension, cardiac hypertrophy, heart failure, and myocardial ischemia-reperfusion injury [1,2,3,4,5,6,7,8,9,10,11]. Currently, the molecular mechanisms underlying its therapeutic actions are still poorly understood, although extensive studies have shown that it affects transcription factors, receptors, channels, kinases, cell survival proteins, growth factors, inflammatory mediators, microRNA, and others [10,12].

H_2_S is an important gaseous mediator, which is synthesized through H_2_S-synthesizing enzymes, cystathionine β-synthase (CBS), cystathionine γ-lyase (CSE), and 3-mercaptopyruvate sulfurtransferase (3MPST) in the mammalian family. It has versatile biological functions, such as vasodilative, cardioprotective, angiogenetic, anti-aging, anti-inflammatory, and antioxidative actions [13,14,15,16,17,18]. H_2_S is a potent antioxidant. It increases cell resistance to oxidative stresses induced by various insults through multiple mechanisms. It scavenges oxygen species, stimulates glutathione (GSH) levels, and enhances the activities of enzymatic antioxidants, SOD, and catalase [16,17]. These effects of H_2_S are mediated by its free thiol activity and its modification on functional proteins through sulfhydration [19,20,21].

Several considerations prompted us to speculate that H_2_S might mediate the pharmacological actions of Tan IIA. First, Tan IIA and H_2_S have many common pharmacological actions. For example, both of them possess vasodilative, anticoagulant, anti-inflammatory, and anti-oxidative actions. Second, both Tan IIA and H_2_S are proved to be effective in treating a variety of cardiovascular diseases with a common pathological basis, such as cell injury, oxidation, and inflammation [1,2,3,4,5,6,7,8,9,10,11,13,14,15,18,22,23,24,25,26], and, third, Tan IIA is the member of the phytoestrogens. It has estrogen-like activity. Some of the pharmacological effects of Tan IIA are via its stimulation on estrogen receptors [22,24,27]. Interestingly, the induction of CSE expression and endogenous H_2_S production by estrogen has been reported [28,29,30]. Therefore, we strongly speculated that stimulation of H_2_S production could be a potential mechanism underlying the pharmacological actions of Tan IIA. The purpose of this study was to test this possibility.

Here, we present our data showing that Tan IIA induced the expression of H_2_S-synthesizing enzyme CSE and stimulated the production of endogenous H_2_S. Furthermore, we analyzed the signaling mechanism involved in the induction of CSE and established the role of H_2_S in protecting cells against oxidative injury. Our study thus characterized H_2_S as a presently unrecognized mechanism contributing to the antioxidative actions of Tan IIA on vascular endothelial cells.

## 2. Materials and Methods

### 2.1. Materials

OxyBlot protein oxidation detection kit was purchased from Merck Millipore (EMD Millipore, Billerica, MA, USA) and SulfoBiotics- HSip-1 DA from Dojindo Laboratories (Kumamoto, Japan). The generation of superoxide anion (O_2_^•^^−^) and ROS were detected using a commercially available kit from Enzo (Tokyo, Japan). Dimedone and acrolein were purchased from Tokyo Chemical Industry (Tokyo, Japan). The anti-cysteine sulfenic acid antibody was from Millipore (Burlington, MA, USA). Beta-cyano L-Alanine (BCA) was from Cayman Chemical (Ann Arbor, MI, USA). Anti-CSE, anti-CBS, and anti-3MPST antibodies were from Santa Cruz Biotechnology, Inc. (Santa Cruz, CA, USA). Anti-actin and anti-P-P38 antibodies, as well as horseradish peroxidase-conjugated anti-rabbit or anti-mouse IgG, were purchased from Cell Signaling, Inc. (Danvers, MA, USA). Alexa 680 Fluor C2 maleimide was from Thermo Scientific (Rockford, IL, USA). 17β-estradiol (E2), Sodium hydrosulfide hydrate (NaHS), L-cysteine hydrochloride, DL-Propargylglycine (PAG), glutathione (GSH), anti-Cx43, and all other chemicals were from Sigma (Tokyo, Japan).

### 2.2. Cell Culture

Human umbilical vein endothelial cells (HUVECs) were obtained from Promo Cell (Heidelberg, Germany) and cultured with endothelial cell growth medium 2 (ready-to-use; Takara-Bio Inc.), supplemented with 5% FBS and 1% antibiotic and antimycotic solution. For experiments, cells were seeded into culture plates and allowed to grow until confluence, followed by stimulation with various reagents for the indicated time intervals.

### 2.3. Western Blot Analysis

Cellular proteins were extracted with 1× SDS lysis buffer (62.5 mM Tris-HCl, 2% SDS, 10% glycerol) that was supplemented with a proteinase inhibitor cocktail or protein kinase inhibitor before use. Protein concentration was measured using the Micro BCA Protein Assay Kit (Thermo Fisher Scientific, Waltham, MA, USA). The same amount of proteins were separated by 10% SDS-PAGE, which was followed by protein transfer to PVDF membranes with wet-blotting apparatus. After treatment of the membrane with 5% skimmed milk or 1% BSA in 0.05% Tween-20 PBS solution (TPBS) for 1 h, the membranes were incubated with primary antibody overnight at 4 °C, followed by washing with TPBS and incubation with peroxidase-conjugated secondary antibody for an additional 1 h. The signal in the membrane was detected using the enhanced chemiluminescence system (Nacalai Tesque, Kyoto, Japan) and captured with a Fujifilm luminescent image LAS-1000 analyzer (Tokyo, Japan). The intensity of the bands was quantified with the NIH ImageJ software (http://rsb.info.nih.gov/ij, accessed on 1 May 2021). The equal loading of sample protein in each lane was confirmed by probing the blot with β-actin or staining with EZ blue.

### 2.4. H_2_S Detection

HUVECs, pretreated with or without Tan IIA, were exposed to H_2_S detection probe Hsip-1 DA (5 µM) and allowed to react for 30 min. After washing, the fluorescent images of cells were captured with a fluorescence microscope (IX71, Olympus, Tokyo, Japan). The cellular fluorescent intensity was quantified using NIH ImageJ software.

### 2.5. Assessment of Protein Carbonylation

The protein carbonylation was determined using a OxyBlot Protein Oxidation Detection Kit (EMD Millipore, Billerica, MA, USA) as described previously [17,31,32]. Briefly, protein samples at the volume of 5 µL at the amount of 5–20 µg were mixed with 5 µL of 12% SDS and 10 µL of DNPH (2,4-dinitrophenylhydrazine) solution for 15 min to denature and derivatize the proteins, respectively. Afterward, 7.5 µL neutralization solution was added, and the samples were subjected to Western blot analysis for carbonylated protein.

### 2.6. Maleimide-Labeling Assay

This assay was performed as we previously reported [31,33]. Cell lysates were extracted and allowed to react with Alexa Fluor 680 C2 maleimide (red fluorescence at the final concentration of 2.5 µM) at 4 °C for 2 h. Afterward, samples were separated by 10% SDS-PAGE and transferred to PVDF membranes. The fluorescent signal in the membranes was captured with a Fujifilm image LAS-1000 analyzer (Fujifilm, Tokyo, Japan) and quantified with ImageJ software. β-actin or EZ blue staining was performed to confirm the equal loading of proteins.

### 2.7. Detection of Sulfhydrated Proteins

To examine the extent of protein sulfhydration, we have used an Alexa Fluor 680-conjugated C2 maleimide assay, reported by Sen et al. [34] with minor modification [19,20,21]. The protocol of the assay includes labeling sulfhydryl (-SH) and sulfhydrated (-SSH) groups of Cys in cellular proteins with fluorescent maleimide, followed by cleavage of the disulfide bonds in (-S-S-Mal) with the reducing chemical DTT. Thus, the loss of the fluorescence intensity (-S-Mal) reflects the extent of sulfhydration. Briefly, cell lysates were allowed to react with Alexa Fluor 680 C2 maleimide (red fluorescence at the final concentration of 2.5 µM) at 4 °C for 2 h. The unlabeled maleimide was removed through the repeated TCA-acetone precipitation. The precipitated proteins were redissolved in 1 x SDS lysis buffer and incubated with or without 1 mM DTT for at least 1 h. The treated samples were subjected to SDS-PAGE separation and transferred to the PVDF membrane. The signal of fluorescent maleimide in the membrane was captured with a Fujifilm image LAS-1000 analyzer (Fujifilm, Tokyo, Japan). β-actin was performed to confirm the equal loading of proteins.

### 2.8. Detection of Sulfenic Acid

Detection of sulfenic acid formation in protein samples was performed using an anti-cysteine sulfenic acid antibody from Millipore (Cat. #07-2139) as we had previously described [33]. Cells at 12-well plate were lysed at 100 μL dimedone lysis buffer (1 mM dimedone, 0.1%TX100, 0.012M Na_2_HPO_4_/0.003M Citric acid, PH6) for 20 min at RT, which was followed by the addition of 25 μL 5 X non-reducing sample buffer that contained 500 μM maleimide. After mixing and centrifuge, 20 μL samples were loaded onto SDS gel. Western blot analysis for sulfenic acids was performed using an anti-dimedone antibody at the dilution of 1:5000.

### 2.9. Detection of ROS Production

The level of superoxide anion (O_2_^•^^−^) and ROS were detected using a kit from Enzo life sciences (Tokyo, Japan, ENZ-51010) following the protocol provided by the manufacturer [32,33,35]. Briefly, cells in 96-well plates were preloaded with O_2_^•^^−^ detection reagent (orange) and oxidative stress detection reagent (green) for 3 h, followed by stimulation with acrolein for 1 h. The fluorescent images of cells were captured using the immunofluorescent microscope (IX71, Olympus, Tokyo, Japan).

### 2.10. Calcein-AM and Propidium Iodide (PI) Staining

Cells were allowed to incubate with a mixture of Calcein-AM (green) and PI (red) solution for 10–20 min. The PI-positive red and calcein-AM positive green cells were photographed using fluorescence microscopy [33,35].

### 2.11. Assessment of Cell Viability with WST Reagent

Cells in 96-well culture plates were incubated with WST reagent (Dojindo, Kumamoto, Japan) for 40–60 min. The optical density (OD) in each well was measured with a spectrometer at the wavelength of 450 nm.

### 2.12. Statistical Analysis

Values are expressed as mean ± SE. A comparison of the two groups was made by Student’s *t*-test. For multiple comparisons with the same control, one-way ANOVA analysis and post hoc comparisons were performed. Both analyses were done using Microsoft Excel (Microsoft, Redmond, WA, USA) or Sigmaplot software. *p* < 0.05 was considered statistically significant.

## 3. Results

### 3.1. Tanshinone IIA Induces CSE Expression and Stimulates H_2_S Production

**Figure 1:**
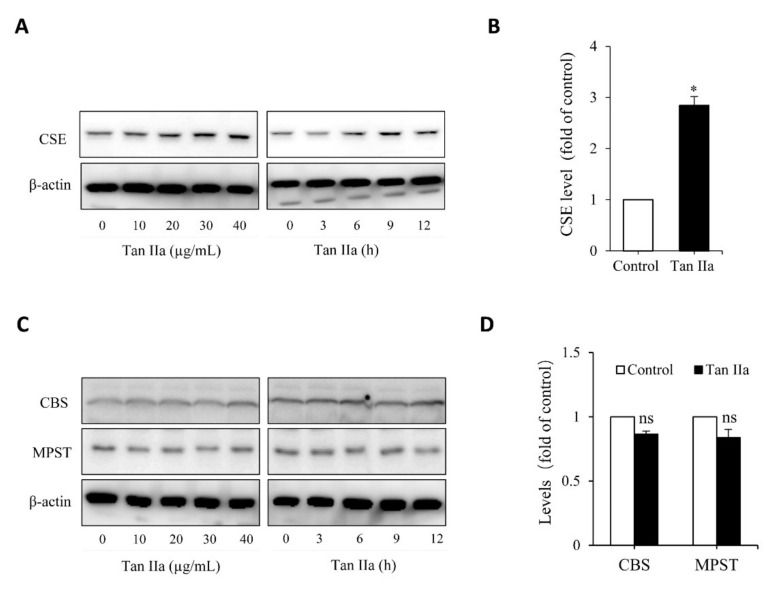
Tan IIA stimulates CSE expression in HUVECs. (**A**,**B**) effects of Tan IIA on the protein level of CSE. Cells were exposed to the indicated concentrations of Tan IIA for 12 h or 20 µg/mL Tan IIA for the indicated times. Cellular lysates were subjected to Western blot analysis for CSE (**A**). The intensity of the bands at the concentration of 20 µg/mL for 12 h was quantified and expressed as the fold of the control (B: mean ± SE, *n* = 3, * *p* < 0.05). (**C**,**D**) Effects of Tan IIA on the protein level of CBS and MPST in HUVECs. The cells were treated the same as above. Cellular lysates were assayed for CBS and MPST (**C**). The densitometric quantitation of the band at 20 µg/mL Tan IIA for 12 h was shown in (**D**). The mark ns denotes no statistical significance.

To determine whether Tan IIA stimulates the production of the endogenous H_2_S, we examined the effect of Tan IIA on H_2_S-synthesizing enzymes in the HUVECs. Figure 1A shows that Tan IIA elevated CSE in a time- and concentration-dependent manner. Treatment of endothelial cells with 20 µg/mL Tan IIA for 6 h led to a significant elevation of CSE (Figure 1B). This effect of Tan IIA was CSE-specific. It did not affect the level of MPST and CBS (Figure 1C,D).

**Figure 2:**
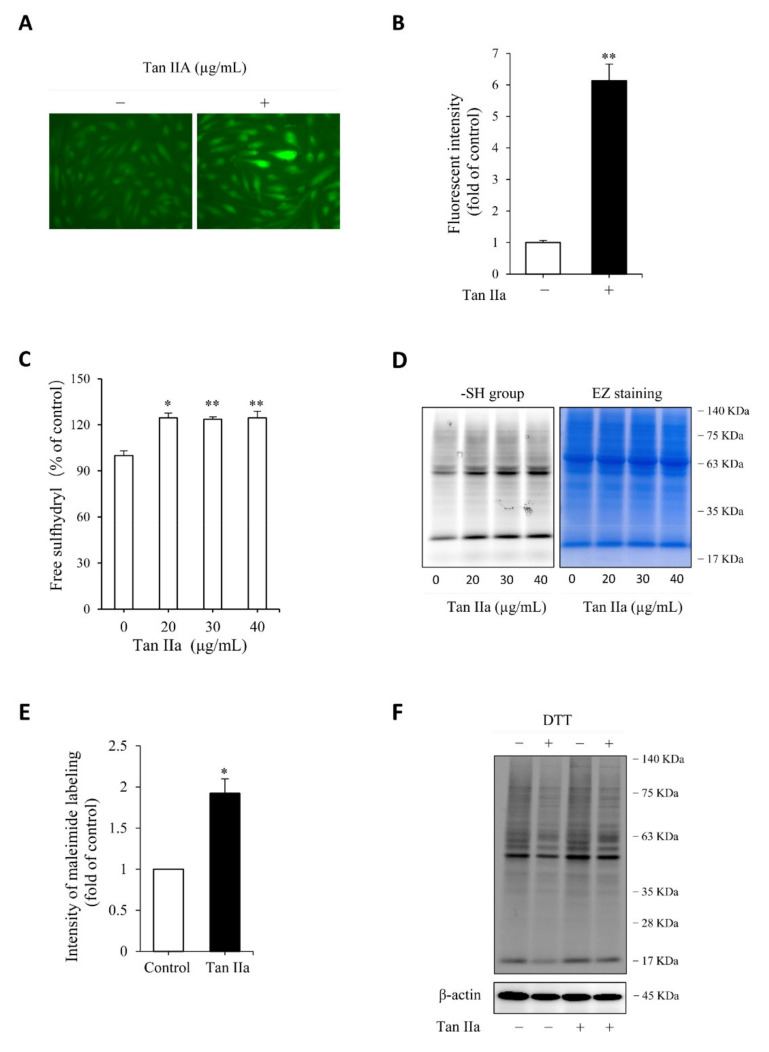
Tan IIA increases endogenous H_2_S production and enhances cellular free thiol activity. (**A**,**B**) effect of Tan IIA on endogenous H_2_S level. Cells pretreated with 20 µg/mL Tan IIA for 8 h were exposed to 5 µM H_2_S detection probe Hsip-1 DA and allowed for a reaction for an additional 30 min. The cellular fluorescence was captured with a fluorescent microscope. Note the enhanced fluorescent intensity of cellular fluorescence in Tan IIA-treated cells (**A**). The cellular intensity of fluorescence was quantified and expressed as fold of control (B: mean ± SE, *n* = 20 cells, ** *p* < 0.01); (**C**) effect of Tan IIA on free sulfhydryl (-SH) activity. Cells were treated with the indicated concentrations of Tan IIA for 8 h. Free SH activity and the amount of -SH groups were detected by Ellman’s reagent (**C**) and maleimide labeling assay (**D**, left panel), respectively. The equal loading of protein in each lane was verified by EZ blue staining (**D**, right panel). The band intensity of fluorescently labeled maleimide at MW around 63 KDa was quantified. The data of control and 30 µg/mL Tan IIA-treated cells in D were shown in (**E**) and expressed as fold of increment relative to control (mean ± SE, *n* = 3;* *p* < 0.05); (**F**) effect of Tan IIA treatment on protein sulfhydrylation. Cells were treated with or without Tan IIA at 30 µg/mL for 8 h. Cellular lysates were allowed to react with 2.5 µM maleimide for 2 h to label -SH groups in proteins. After removing unlabeled maleimide, the samples were treated with 1 mM DTT for 1 h to cleave the disulfide bond in -S-S-Mal groups, followed by SDS-PAGE to separate the protein and detection of fluorescence in the gel. The loss of fluorescence after reductive treatment indicates the presence of sulfhydrylated (-SSH) proteins. Note the reduced fluorescent intensity after DTT treatment in both control and Tan IIA-treated samples.

We then proceeded to test whether the elevated CSE was associated with an increased level of endogenous H_2_S. For this purpose, we have used a cell membrane permeable H_2_S probe Hsip-DA, a chemical that enables fluorescent imaging of H_2_S. Figure 2A,B show that Tan IIA-pretreated cells exhibited a much stronger cellular fluorescence than control cells, indicating a higher level of endogenous H_2_S.

H_2_S has reductive activity. It maintains Cys residues at the reduced state and induces protein sulfhydrylation, a post-translational modification made by H_2_S through adding additional sulfur to sulfhydryl (-SH) groups of Cys in proteins [15,19,21,31]. We, therefore, tested the effect of Tan IIA on free sulfhydryl (-SH) activity and protein sulfhydrylation (-SSH). Detection of -SH activity using Ellman’s reagent (Figure 2C) and maleimide-labeling assay (Figure 2D,E) revealed that Tan IIA stimulated -SH activity and elevated the number of -SH groups. Because the increased -SH activities include both -SH groups and sulfhydrated Cys (-SSH), we, therefore, confirmed the presence of sulfhydrated proteins. For this purpose, maleimide-prelabeled cellular proteins were treated with the reductive chemical DTT. The loss of the fluorescent signal after DTT treatment indicated the presence of -SSH groups in proteins. Figure 2F shows that DTT-treated samples displayed an obviously reduced intensity of several bands at a wide range of MWs, confirming the existence of protein sulfhydrylation.

Collectively, these observations indicate that Tan IIA induced CSE expression, stimulated H_2_S production, and enhanced cellular thiol activity.

### 3.2. cAMP Pathway Mediates Tan IIA-Induced Elevation of CSE

**Figure 3:**
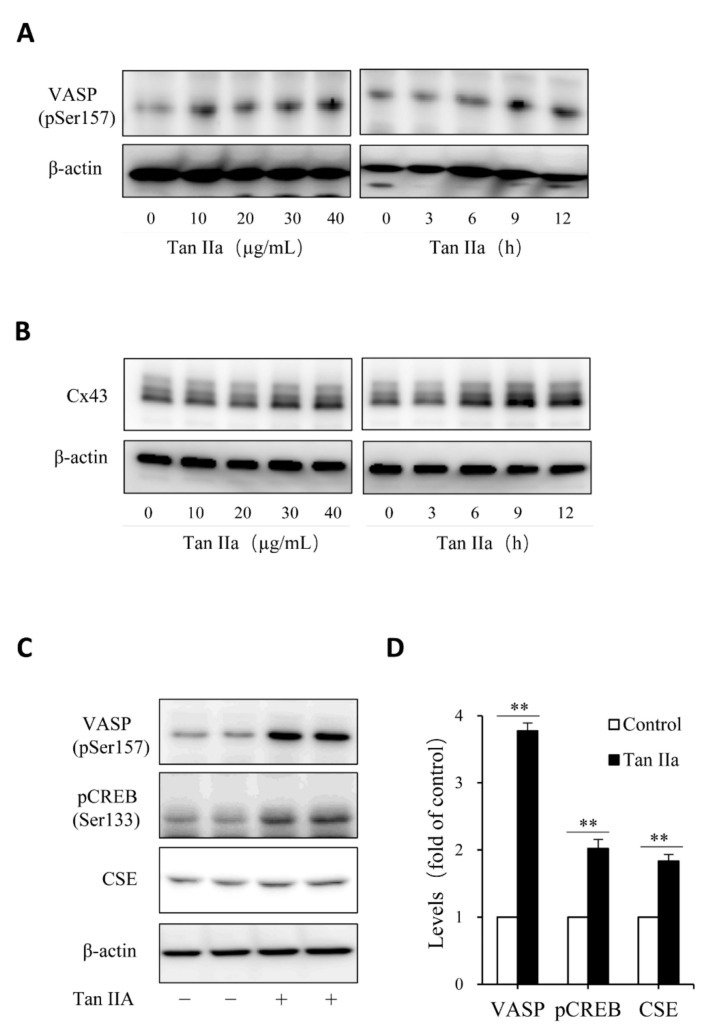
Tan IIA activates the cAMP signaling pathway. (**A**–**D**) effects of Tan IIA on VASP protein phosphorylation and Cx43 expression. Cells were exposed to the indicated concentrations of Tan IIA for 12 h or 20 µg/mL Tan IIA for the indicated times. Cellular lysates were extracted and subjected to Western blot analysis for the level of VASP phosphorylation and Cx43 expression; (**C**,**D**) effects of Tan IIA on the activation of the PKA signaling pathway. The cells were treated with 30 µg/mL Tan IIA for 12 h. Cellular lysates were assayed for the level of VASP and CREB phosphorylation, as well as CSE expression (**C**). The densitometric quantitation of VASP, CREB, and CSE was shown in (**D**). Data shown are mean ± SD (** *p* < 0.01; *n* = 3).

**Figure 4:**
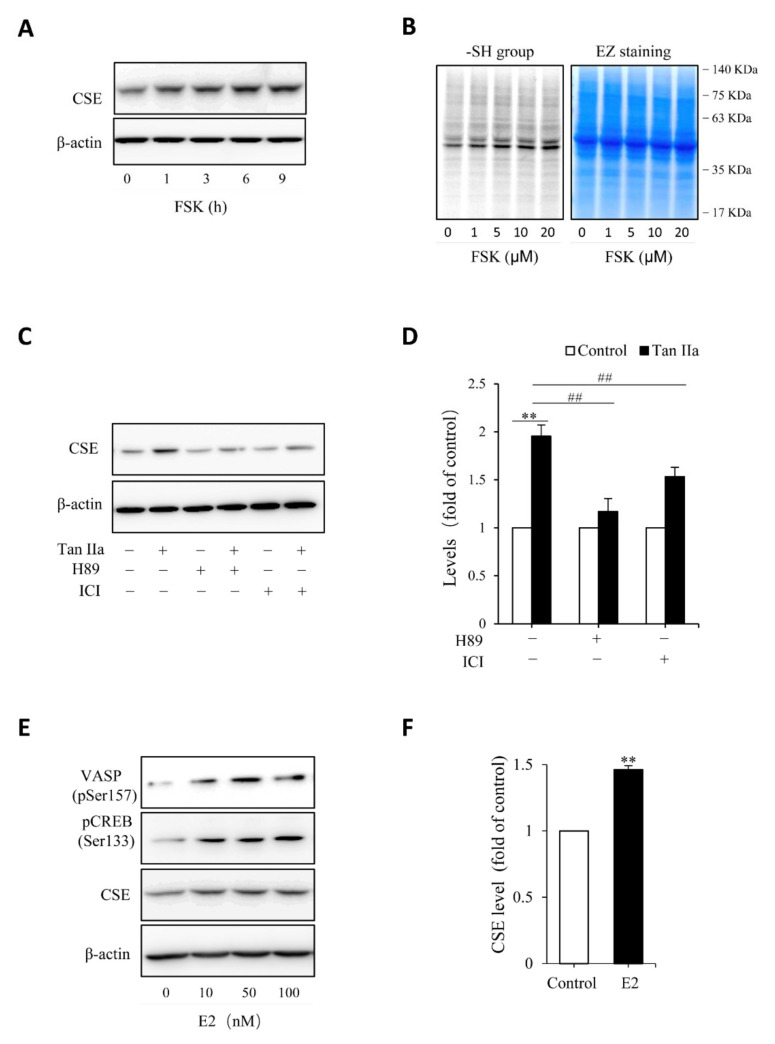
Estrogen receptor and cAMP signaling pathway mediate Tan IIA-induced CSE expression. (**A**,**B**) stimulation of CSE expression and -SH activity by PKA activator forskolin (FSK). HUVECs were incubated with 10 µM FSK for the indicated time intervals (**A**) or the indicated concentrations of FSK for 6 h (**B**). Cellular lysates were extracted and subjected to Western blot analysis for CSE (**A**) or maleimide labeling assay for -SH groups (**B**, left panel). The equal loading of protein in each lane was verified by probing the blot with β-actin or EZ blue staining (**B**, right panel); (**C**,**D**) effects of PKA inhibitor and ER receptor blocker on Tan IIA-induced CSE expression. Cells were exposed to 30 µg/mL Tan IIA in the presence or the absence of 10 µM PKA inhibitor H89 or 100 µM estrogen receptor blocker ICI for 12 h. Cellular lysates were subjected to Western blot analysis of CSE. The densitometric analysis of the blot in (**C**) is shown in (**D**). Data shown are mean ± SE, *n* = 3. ** *p* < 0.01 versus control; ^##^ *p* < 0.01 versus Tan IIa alone; (**E**,**F**) activation of PKA and induction of CSE by β-Estradiol (E2). HUVECs were incubated with different concentrations of E2 for 12 h. Cellular lysates were extracted and subjected to Western blot analysis for the level of VSAP and CREB phosphorylation and CSE expression. The densitometric analysis of the bands of CSE in (**E**) is shown in (**F**). Data shown are mean ± SE, *n* = 3. ** *p* < 0.01.

Given that Tan IIA has vasodilative actions and it activates the vasodilative cAMP signaling pathway [36,37], while the pathway has been implicated in the regulation of CSE [38,39], we, therefore, tested the possible participation of this pathway. To this end, we examined the phosphorylated level of PKA substrates, VASP and CREB, and detected the changes of CREB-controlled gene product, Cx43 [40]. Figure 3 shows that Tan IIA elevated VASP and CREB phosphorylation and increased Cx43 expression, indicating an activation of the PKA pathway. To determine the role of this pathway, we activated this pathway with adenyl cyclase activator forskolin and found that forskolin reproduced the CSE-elevating effect of Tan IIA (Figure 4A). It induced a concentration-dependent elevation in CSE. In addition, it also elevated thiol activity (Figure 4B). Inhibited PKA with specific PKA inhibitor H89 largely abolished the CSE-elevating effect of Tan IIA (Figure 4C,D). These results indicate that Tan IIA induced CSE expression via activation of the cAMP signaling pathway.

Because some of the actions of Tan IIA are mediated by estrogen receptor (ER) [22,24,27], we, therefore, also tested its involvement. Figure 4C shows that ER inhibitor ICI also prevented the CSE elevation. In further support of the role of ER signaling pathway, we found that stimulation of endothelial cells with estrogen mimicked the CSE-elevating action of Tan IIA (Figure 4E,F). Moreover, it was also associated with increased phosphorylation of PKA substrate VASP and CREB (Figure 4E). Collectively, these observations indicate that Tan IIA induced CSE expression via ER and cAMP signaling pathway.

### 3.3. Acrolein Induces Oxidative Endothelial Cell Injury

**Figure 5:**
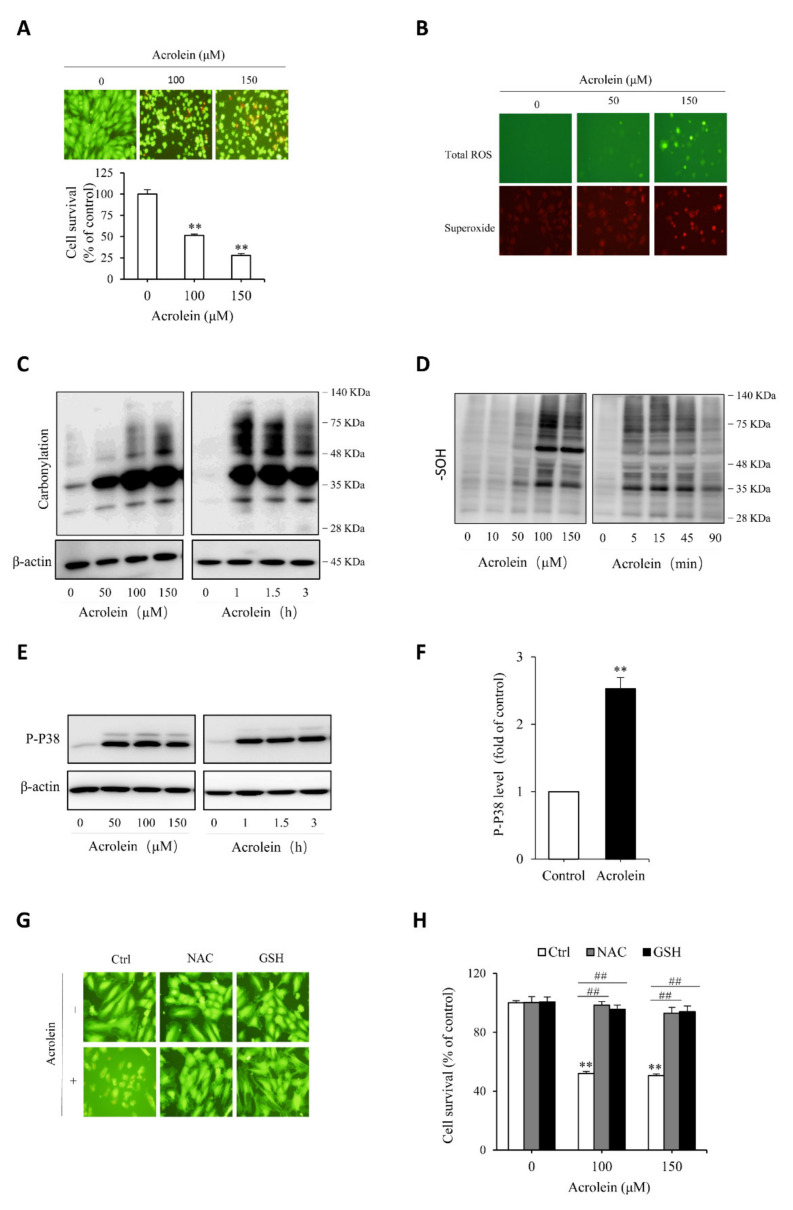
Acrolein induces oxidative endothelial cell injury. (**A**) effect of acrolein on endothelial cell viability. HUVECs were exposed to the indicated concentrations of acrolein for 6 h. Afterward, cells were stained with Calcein-AM/PI or assayed for cell viability with a WST reagent. Data shown in the graph are mean ± SE (*n* = 4). ** *p* < 0.01 versus control; (**B**) induction of ROS generation by acrolein. Cells were treated with 50 µM acrolein for 1 h and assayed for the intracellular level of superoxide (red) and total ROS (green) with a kit from Enzo; (**C**–**F**) induction of protein carbonylation, sulfenic acid formation and P38 phosphorylation by acrolein. HUVECs were incubated with the indicated concentrations of acrolein for 3 h (**C** and **E**) or 90 min (**D**), or 100 µM acrolein for the indicated time. Cellular lysates were subjected to Western blot analysis. (**F**) Densitometric analysis of P38 activation under the stimulation of 100 µM acrolein for 3 h. Data shown are mean ± SE (*n* = 3) ** *p* < 0.01; (**G**,**H**) prevention of acrolein-induced cell death by thiol antioxidants. Cells were exposed to either 150 µM acrolein (**G**) or the indicated concentrations of acrolein (**H**) in the presence or absence of 1 mM GSH or NAC for 6 h. The cell viability was determined using Calcein AM/PI staining (**G**) and WST assay (**H**). Data shown in H are mean ± SE (*n* = 4). ** *p* < 0.01 versus control; ^##^ *p* < 0.01 versus acrolein alone.

To test the protective role of Tan IIA on oxidative endothelial cell injury, we have used acrolein as an oxidative inducer. As an unsaturated aldehyde, acrolein plays a vital role in the initiation, maintenance, and exaggeration of oxidative stress. It has been reported to exert many pathological effects on endothelial cells [41,42,43]. First, we confirmed the cytotoxicity of acrolein on endothelial cells. Figure 5A shows that acrolein induced a concentration-dependent endothelial cell injury, as indicated by the cell shape change, increased the number of PI-positive dead cells, and decreased formazan formation. Then, we determined the role of oxidative stress in mediating acrolein-induced cell injury. Figure 5B shows that acrolein stimulated superoxide and ROS production, as evidenced by the increased intensity of cellular fluorescence, positively correlated with the intracellular level of superoxide (red) and ROS (green). Consistently, other oxidative indicators, such as protein carbonylation, sulfenic acid formation, and P38 phosphorylation, were all increased by acrolein (Figure 5C–F). All of these results indicate that acrolein induced oxidative stress in HUVECs. To establish the role of oxidative stress in cell injury, we supplemented cells with the thiol-antioxidant GSH or its precursor NAC. This treatment completely prevented the cell injury (Figure 5G,H). Collectively, these observations indicate that acrolein induced oxidative endothelial injury.

### 3.4. Tan IIA Prevents Acrolein-Initiated Oxidative Endothelial Cell Injury

**Figure 6:**
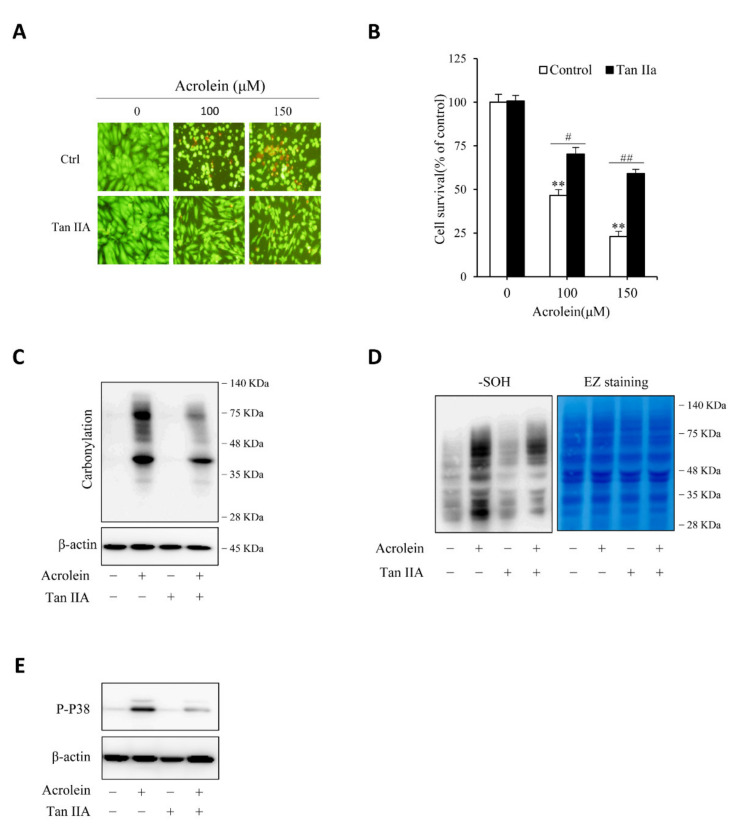
Tan IIA attenuates acrolein-elicited oxidative endothelial injury. (**A**,**B**) effect of Tan IIA on acrolein-induced cell injury. HUVECs were treated with the indicated concentrations of acrolein in the presence or absence of 30 µg/mL Tan IIA for 12 h. The cell viability was determined by Calcein-AM/PI staining (**A**) and WST assay (**B**). Data in (**B**) are expressed as mean ± SE, *n* = 4. ** *p* < 0.01 versus zero control; # *p* <0.05, ## *p*< 0.01 versus respective acrolein alone. (**C**–**E**) effect of Tan IIA on acrolein-induced protein carbonylation, sulfenic acid formation and P38 activation. HUVECs were pretreated with or without 30 µg/mL Tan IIA for 12 h, followed by exposure to 100 µM acrolein for an additional 1 h. Cellular lysates were assayed for protein carbonylation (**C**), sulfenic acid formation (**D**, left blot), and P38 activation (**E**). Equal loading of protein in each lane was confirmed by probing the blot with β-actin or EZ blue staining (**D**, right blot).

We then proceeded to determine the effect of Tan IIA on acrolein-induced cell injury. Figure 6A,B show that Tan IIA significantly prevented acrolein-induced cells injury, as indicated by the improved cell shape, decreased number of PI-positive red cells, as well as enhanced cell viability. This effect of Tan IIA was associated with a reduced level of protein carbonylation, sulfenic acid formation, and P38 activation (Figure 6C–E), suggesting inhibition of oxidative stress.

Other than acrolein, we also observed that Tan IIA effectively prevented H_2_O_2_-induced endothelial cell injury (data not shown), an effect that has been previously described by Lin et al. [44].

### 3.5. H_2_S Contributes to Endothelial Defense to Oxidative Cell Injury

**Figure 7:**
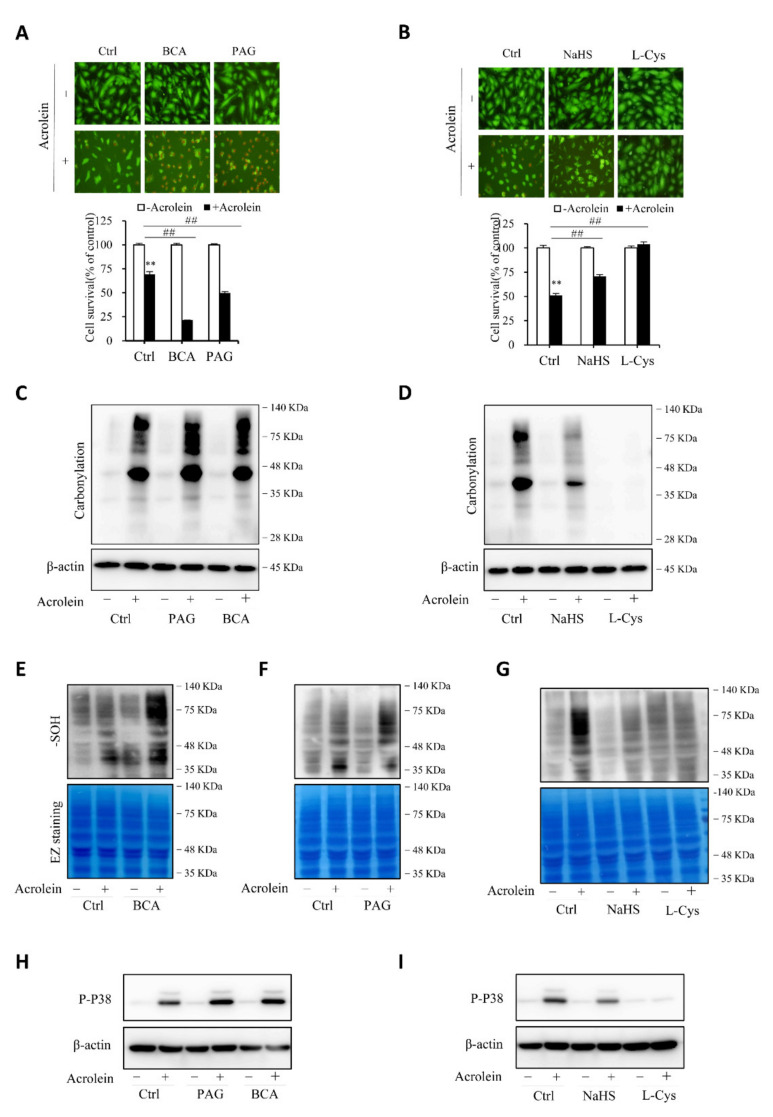
H_2_S contributes to the cellular defense against acrolein-initiated oxidative cell injury. (**A**,**B**) effects of H_2_S on acrolein-initiated cell death. HUVECs were exposed to 150 µM acrolein in the presence or absence of 2 mM BCA or 3 mM PAG (**A**), or 1 mM NaHS or 2 mM L-cysteine (L-Cys). The cell viability was determined by Calcein-AM/PI staining (**A**) and WST assay (**B**). Graph below the images in A and B are mean ± SE, *n* = 4; ** *p* < 0.01 versus control, ^##^ *p* < 0.01 versus acrolein alone. (**C**–**I**) effect of H_2_S on acrolein-induced protein carbonylation, sulfenic acid formation, and P38 activation. HUVECs were exposed to 75 µM acrolein in the presence or absence of 2 mM BCA, 3 mM PAG, 1 mM NaHS or 2 mM L-cysteine for 1 h. Cellular lysates were assayed for protein carbonylation (**C**,**D**), sulfenic acid formation (**E**–**G**), and P38 activation (**H**,**I**). Equal loading of protein in each lane was confirmed either by re-probing the same blot with β-actin or through EZ blue staining.

To establish the role of H_2_S in the protection of cells against acrolein-induced oxidative stress, we compared cell responses to acrolein with or without endogenous or exogenous H_2_S. Figure 7A,B show that inhibition of CSE with CSE inhibitors, BCA, and PAG, sensitized cells to the cytotoxicity of acrolein, whereas the supplement of cells with exogenous H_2_S donor NaHS or L-cysteine prevented cell injury. Consistently, the level of oxidative stress, as indicated by protein carbonylation, sulfenic acid formation, and P38 activation, was potentiated after inhibiting endogenous H_2_S production and attenuated by supplementing exogenous H_2_S (Figure 7C–I). The observations indicate that endogenous H_2_S plays a pivotal role in endothelial defense again acrolein-initiated oxidative cell injury.

## 4. Discussion

Tan IIA is a commonly-used drug for the treatment of cardiovascular diseases. It has a broad range of pharmacological functions [1,2,3,4,5,6,7,8,9,10,11]. This study found that Tan IIA elevated CSE and stimulated endogenous H_2_S production in cultured HUVECs. Given that Tan IIA and H_2_S have many similarities in their pharmacological actions, induction of H_2_S could be an essential mechanism by which Tan IIA exerts its therapeutic effects.

H_2_S production in mammalian cells is controlled by three major H_2_S-synthesizing enzymes [15]. Herein, we demonstrated that Tan IIA specifically upregulated CSE without significant influence on MPST and CBS, indicative of a distinct regulation. The significantly increased level of endogenous H_2_S after Tan IIA stimulation and dramatic changes in the redox state following CSE inhibition suggested that CSE was the predominant enzyme responsible for H_2_S production and function in HUVECs. Our observations are consistent with the current view that H_2_S-producing enzymes have a distinct pattern of responses to various stimuli and that CSE plays a predominant role in endothelial cells [45,46]. Currently, the reason for the selective upregulation of CSE is unclear, but it should be related to the specific actions of Tan IIA on CSE-regulating mechanisms.

Our study characterized the cAMP pathway as the signaling mechanism mediating Tan IIA-induced CSE elevation. As a vasodilator, activation of the central vasodilative signal by Tan IIA is not surprising [24]. It is reported that Tan IIA activates the cAMP pathway in melanocytes [36]. It appeared that this also held in endothelial cells. Several studies have implicated cAMP signaling in regulating CSE expression and activity via mechanisms involving its action on CSE gene transcription and protein modification. CREB binding sites in the promoter region of CSE have been reported [38], and the recruitment of CREB activated the CSE gene and facilitated CSE transcription. More recently, Liu et al. described that PKA-mediated post-translational modification of CSE via O-GlcNacylation enhanced the CSE level and activity [39]. These mechanisms may underlie the stimulating effect of Tan IIA on the CSE/H_2_S level.

How did Tan IIA activate the cAMP signaling pathway? It was most likely through its action on ER signaling pathway. As a phytoestrogen, Tan IIA has estrogen-like activities, which mediates many pharmacological effects of Tan IIA [22,27,47,48]. Our study indicates that this pathway also mediated the elevation of CSE. This conclusion is supported by the observations that, in line with previous reports [28,29,30,49], estrogen also induced CSE expression in HUVECs and that the CSE-elevating effect of Tan IIA could be blocked by ER inhibitors. Of note, as a downstream signal of G protein-coupled estrogen receptor alpha, activation of the cAMP pathway by estrogen has been extensively documented [50,51]. However, the direct evidence supporting cAMP signaling in the estrogen-induced elevation of CSE has not been reported. In clarifying the molecular mechanism of Tan IIA, we accidentally found that estrogen also worked through the same pathway. Thus, our study also provided novel mechanistic insight into the CSE-elevating action of estrogen.

It is also worth mentioning that, other than the cAMP signaling pathway, Tan IIA may also regulate CSE via different routes. For example, Tan IIA has been shown to induce nitric oxide (NO) [24], a vasodilator that activates the cGMP signaling pathway. Recently, the upregulation of CSE activity by PKG via the direct phosphorylation of CSE (nongenomic) has also been reported [30,49]. Moreover, our group and others have demonstrated the existence of close crosstalk between PKG and PKA signaling pathways [40,52]. The involvement of these mechanisms in the upregulation of CSE is also possible.

Our study demonstrated that stimulation of endogenous H_2_S contributed to the antioxidative actions of Tan IIA on endothelial cells. The antioxidative effects of H_2_S have been extensively documented [15,53]. H_2_S scavenges ROS and increases cellular defense against oxidative stress. It induces several enzymatic antioxidants via activation of Nrf2 [17]. In addition, it also increases the thiol antioxidant level and maintains thiol at a reductive state [16,19,21,31]. We have recently reported that H_2_S also suppresses the thioredoxin/ASK1/P38 redox signaling pathway [31]. Intriguingly, most of the reported antioxidative effects of H_2_S have also been reported in Tan IIA [5,7,8,10]. Herein, we demonstrated that Tan IIA and H_2_S similarly suppressed ROS-mediated protein modification and redox signaling activation. Together with the worsened redox status and cell survival after the inhibition of endogenous H_2_S, these observations support the notion that induction of H_2_S mediated the antioxidative action of Tan IIA.

We have used acrolein to induce oxidative stress. The rationale for this selection is multifold. First, acrolein is a ubiquitous pollutant present in the environment, food, and water. It is also produced from endogenous metabolic activity [54]. Human exposures to acrolein are common. The adverse effects of acrolein on human health, especially its effects on the vascular system, have also been well documented [41,55]. It is more relevant to pathological situations. Second, acrolein is especially insidious in oxidative stress. It not only initiates oxidative stress but also potentiates oxidative stress via the vicious cycle “acrolein-ROS-lipid peroxidation-acrolein” [56]. Third, acrolein induces oxidative stress via multiple mechanisms, including stimulating ROS production and suppressing the cellular antioxidative defense system. Lastly, thiol antioxidants are considered to be the central defense mechanism against acrolein toxicity. H_2_S, on the one hand, has thiol activity that can directly scavenge acrolein; on the other hand, it also upregulates GSH synthesis and maintains thiol in the reduced state [16,31,53]. Therefore, acrolein-induced oxidative cell injury should be an excellent model to demonstrate the antioxidative actions of H_2_S and Tan IIA. Of note, other than acrolein, we also confirmed the previous reports that Tan IIA and H_2_S prevented cell death induced by H_2_O_2_ in endothelial cells (data not shown) [57].

Our findings could have significant implications. First, our study revealed a presently unreported mechanism by which Tan IIA exerts its therapeutic effects. Given that H_2_S and Tan IIA share many common pharmacological actions on vascular systems, such as vasodilation, anticoagulation, and antiinflammation, it is conceivable that, apart from the antioxidative effect as demonstrated in this study, H_2_S also contributed to the other pharmacologic actions of Tan IIA. Second, our study indicated that Tan IIA and H_2_S could be used to prevent and treat acrolein-induced toxicity. Currently, thiol antioxidants are considered to be the most effective way for acrolein detoxification [54]. Stimulation of the production of endogenous H_2_S and other thiol-active antioxidants by Tan IIA makes Tan IIA an ideal drug to prevent acrolein toxicity. Third, H_2_S deficiency has been implicated in aging, Alzheimer‘s disease, cardiovascular, and metabolic disorders [13,14,30]. Stimulating endogenous H_2_S production via the upregulation of H_2_S-producing enzymes could be a promising therapeutic approach to prevent and treat these diseases. In this context, pharmacological inducers like Tan IIA could have widespread applications.

## 5. Conclusions

Our study characterized H_2_S as a presently unknown molecule mechanism mediating the antioxidative actions of Tan IIA. H_2_S has multifaced biological functions and H_2_S deficiency has been implicated in many pathological situations, and induction of H_2_S via upregulation of the H_2_S-synthesizing enzyme by Tan IIA could be exploited to treat various diseases.

## Data Availability

The data is contained within the article.
